# LINC01234 Accelerates the Progression of Breast Cancer via the miR-525-5p/Cold Shock Domain-Containing E1 Axis

**DOI:** 10.1155/2022/6899777

**Published:** 2022-07-25

**Authors:** Jia Yu, Xinxin Zhang, Rongfang He, Xiaoyan Yang, Juan Hu, Fang Tan, Jinyu Yao, Xiaoyong Lei, Gebo Wen

**Affiliations:** ^1^Hunan Province Key Laboratory of Tumor Cellular & Molecular Pathology, Cancer Research Institute, Hengyang Medical College, University of South China, Hengyang, Hunan, China; ^2^Hunan Province Cooperative Innovation Center for Molecular Target New Drug Study, Hunan Provincial Key Laboratory of Tumor Microenvironment Responsive Drug Research, Institute of Pharmacy and Pharmacology, University of South China, Hengyang, Hunan, China; ^3^State Key Laboratory of Oncology in South China, Sun Yat-sen University Cancer Center, Guangzhou, Guangdong, China; ^4^Department of Pathology, The First Affiliated Hospital of University of South China, Hengyang, China

## Abstract

**Backgrounds:**

Long noncoding RNAs (lncRNAs) are strongly associated with the development of breast cancer (BC). As yet, the function of LINC01234 in BC remains unknown.

**Methods:**

Using biological information, the potential lncRNA, miRNA, and target gene were predicted. LINC01234 and miR-525-5p expression in BC tissues was detected using quantitative real-time reverse transcription polymerase chain reaction. Fluorescence in situ hybridization was used to determine the distribution of LINC01234. Cell proliferation was analyzed using CCK-8 assay, colony formation, terminal deoxynucleotidyl transferase dUTP nick end labeling staining, and apoptosis evaluated using flow cytometry. Western blotting was used to evaluate protein expression. Dual-luciferase® reporter, RNA pull-down, and RNA immunoprecipitation assays were performed to analyze the binding relationships among LINC01234, miR-525-5p, and cold shock domain-containing E1 (CSDE1).

**Results:**

We screened out LINC01234, found to be significantly increased in BC tissues, associated with a poor prognosis, and positively correlated with tumor size of BC. Knockdown of LINC01234 suppressed BC cell growth and facilitated apoptosis. Dual-luciferase reporter®, RNA pull-down, and RNA immunoprecipitation assays confirmed that LINC01234 and CSDE1 directly interacted with miR-525-5p. Upregulation of miR-525-5p and suppression of CSDE1 inhibited BC cell growth and induced cell apoptosis.

**Conclusion:**

Upregulation of LINC01234 contributes to the development of BC through the miR-525-5p/CSDE1 axis. LINC01234 may be one of the potential diagnostic and treatment targets for BC.

## 1. Introduction

Breast cancer (BC) is one of the most commonly diagnosed cancers worldwide and is associated with high mortality. In 2020, BC surpassed lung cancer for the first time, causing more than 2.1 million cases and 680,000 deaths in 2020 alone. Among women, BC ranks the first in terms of incidence and mortality in most countries, accounting for nearly 24.5% of all cancer cases and 15.5% of cancer-related deaths [1]. The mortality rate of BC remains high owing to abnormal cell proliferation and distant metastasis of BC cells [2, 3]. Despite the emergence of multiple combination therapies for improving the quality of life and overall survival of patients with BC, a few patients nonetheless experience recurrence and metastasis. BC is a heterogeneous tumor with complex molecular alterations [4]. Hence, it is important to understand the potential underlying molecular mechanisms and investigate potential prognostic and therapeutic targets.

lncRNAs are associated with a variety of pathological and physiological processes [5, 6], especially cancers [7–9]. Several lncRNAs can act as tumor suppressor genes or oncogenes to influence BC progression [10, 11]. Importantly, lncRNAs can function as competitive endogenous RNAs (ceRNAs) that adsorb miRNAs, regulating downstream gene expression in BC [12–15]. As a molecular sponge, lncRNAs adsorb and separate miRNA from target mRNA, thus affecting mRNA translation. lncRNAs thus possess considerable potential as biomarkers for tumor diagnosis, prognostic evaluation, and targeted treatment formulation [16]. LINC01234, a newly discovered lncRNA, is located on chromosome 12q24.13. Previous studies have shown an upregulation of LINC01234 in ovarian cancer [17], liver cancer [18], and triple-negative breast cancer (TNBC) [19]. However, both the biological impact and mechanism of LINC01234 in the pathogenesis of BC are scarcely known.

miRNAs, belonging to a class of noncoding RNAs, are single-stranded RNAs with 19–25 nucleotides and are known to play an important role in cancers [20]. In general, miRNAs exert their effects via base complementary pairing with target mRNAs, degrading the corresponding mRNAs, or suppressing mRNA translation [21]. miRNA is the core element in the ceRNA network, considered to be a novel mechanism between RNAs. A low expression of miR-525-5p has been found in cancers, including lung cancer [22], thymic carcinoma [23], and BC [24]. Thus, as a potential tumor suppressor, miR-525-5p is believed to be crucial in the pathogenesis of multiple types of cancers.

Recently, remarkable progress has been achieved with the development of high-throughput sequencing technologies and public databases, which have enabled researchers to identify tumor-driving genes and the relevant signaling pathways related to tumor progression [25–27]. In this study, we screened out LINC01234 based on datasets from The Cancer Genome Atlas (TCGA) databases. We verified that LINC01234 expression levels were increased in cancer tissues and BC cell lines and that upregulation of LINC01234 was associated with poor prognosis of BC. We also examined the function of LINC01234 in BC cells and identified that its suppression could reduce cell proliferation and promote apoptosis. Furthermore, LINC01234 was found to function as a ceRNA in accommodating CSDE1 expression by adsorbable sponging miR-525-5p, thereby suggesting the possibility of using LINC01234 as one of the potential biomarkers and targets for BC diagnosis and treatment.

## 2. Materials and Methods

### 2.1. Identification of Differentially Expressed lncRNAs

The TCGA-BRCA lncRNA data were obtained from Genomic Data Commons (GDC) Data Portal (https://portal.gdc.cancer.gov/). The data so obtained were normalized using the rate monotonic algorithm. Differentially expressed lncRNAs (DElncRNAs) were identified using differential expression analysis, utilizing the DESeq2 package of R (The R Foundation for Statistical Computing, Vienna, Austria). The cutoff criteria applied were *p* < 0.05 and logFC > 2. Volcano plots and heat maps were constructed using the “ggplot2” and “pheatmap” packages in R, respectively. The correlation of gene expression was analyzed using the “cor.test” function. The Kaplan–Meier survival curve for DElncRNAs was plotted using the “survival” package in R.

### 2.2. Prediction of Target Genes

The interaction between lncRNA and miRNA was analyzed through miRcode (http://mircode.org), StarBase (http://starbase.sysu.edu.cn/index.php), and lncBasev2.0 (http://carolina.imis.athena-innovation.gr/diana_tools/web/index.php?r=lncbasev2%2Findex-predicted). Moreover, the interaction between miRNA and mRNA was analyzed utilizing miRDB (http://mirdb.org), miRTarBase (http://mirtarbase.cuhk.edu.cn), and TargetScan (http://www.targetscan.org). A Venn diagram was drawn to identify the overlapping target miRNAs and mRNA.

### 2.3. Clinical Sample Collection and Analysis

BC and adjacent normal tissue samples were acquired from 101 patients during surgery, prior to their receiving chemotherapy or radiotherapy at the First Affiliated Hospital of University of South China, from March 2019 to January 2021. The BC and normal tissue samples were collected and frozen in liquid nitrogen immediately. The study was approved by the Ethics Committee of the First Affiliated Hospital of University of South China (No. 2019LL0912001).

### 2.4. Immunohistochemical Analysis

The expression levels of CSDE1 protein in BC tissues were detected by immunohistochemistry (IHC). Anti-CSDE1 (Abcam, ab201688) was used for IHC, as per a previously reported method [28]. IHC staining was captured under microscopy, and IHC optical density scores were calculated to quantify protein expression [29].

### 2.5. Cell Culture

Human normal breast epithelial cell line (MCF-10A, CL-0525) and BC cell lines (MDA-MB-231 (CL-0150), SK-BR-3 (SKBR-3, CL-0211), MCF-7 (CL-0149), HS-578T (CL-0114), MDA-MB-468 (CL-0290), T-47D (CL-0228), and BT-474 (CL-0040)) were obtained from Procell (Wuhan, China); BC cell lines (HCC1806 (CRL-2335) and BT-474 (HTB-20)) were acquired from ATCC (Rockville, USA) and cultivated in the recommended media. All cell lines were subcultured in our laboratory for less than six months.

### 2.6. qRT-PCR

The total RNA was isolated by TRIzol (#9109, TAKARA, Japan) and dissolved in diethyl pyrocarbonate-treated ddH_2_O (DEPC, #6079, MACKLIN, Shanghai). Bestar™ qPCR RT Kit (#2220, DBI Bioscience, Ludwigshafen, German) was utilized for synthesizing cDNA. The expression of target genes was determined using Bestar™ qPCR MasterMix (#2043, DBI Bioscience) with a real-time fluorescence quantitative polymerase chain reaction (PCR) instrument (Bio-Rad, CFX96, USA). U6 and GAPDH were set as internal references. The 2^-△△Ct^ method was employed for the calculation of certain gene expressions. The primers used are detailed in Table 1.

### 2.7. Cell Transfection

The synthesis of small-interfering RNA (si-RNA) targeting LINC01234 (sh-LINC01234, 5′-AACAUUCAUCUCAGAUUCCUGdTdT-3′) and CSDE1 (sh-CSDE1, 5′-AUAUCUCUUUUACAACAUCACdTdT-3′), miR-525-5p mimics, miR-525-5p inhibitors, as well as the overexpressing plasmids of pcDNA3.1-LINC01234, pcDNA3.0-CSDE1, psi-Check-LINC01234-WT, psi-Check-LINC01234-MUT, psi-Check-3′UTR-CSDE1-WT, and psi-Check-3′UTR-CSDE1-MUT was all completed by Synbio Technologies (Suzhou, China). Cells were plated into six-well plates overnight (5 × 10^5^/ml, 2 ml/well) and transfected using Lipofectamine™ 3000 reagent (Invitrogen). After transfection for 48 hours, cells were collected for the subsequent experiments. The cell transfection efficiency was determined using quantitative real-time reverse transcription (qRT-PCR).

### 2.8. CCK-8 Assay

BC cells were inoculated into 96-well plates (1 × 10^4^/ml, 100 *μ*l/well) and transfected for 48 h. Cells were cultured for the indicated times. A total of 10 *μ*l of CCK-8 solution (Dojindo, Japan) was added to each well. After 2 hours, the absorbance at 450 nm was measured using a microplate reader (BioTek, Epoch, USA).

### 2.9. Colony Formation Assay

Transfected BC cells (800 cells per well) were seeded into a 35 mm petri dish and incubated in the complete medium for two weeks until visible clones appeared. After rinsing with phosphate-buffered saline (PBS), cells were immobilized in 4% paraformaldehyde (Aladdin, Shanghai, China) for 30 min and stained with 10% crystal violet (Aladdin) for 10 min. Finally, the clones were captured and counted under a microscope.

### 2.10. Cell Apoptosis Assay

The Annexin V-FITC/PI staining kits (Beyotime, Shanghai, China) were employed to analyze the extent of cell apoptosis. A single-cell suspension of cells was created using 0.25% trypsin (Genview, Beijing, China) and centrifuged at 1000 rpm. After rinsing with PBS, the cells were resuspended and stained with FITC-labeled Annexin V (10 *μ*l) and PI reagent (5 *μ*l) mix. The mixture was kept in the dark for 10 min. Finally, cell apoptosis was analyzed using a flow cytometer (Becton Dickinson, FACSCalibur, USA).

### 2.11. Terminal Deoxynucleotidyl Transferase Biotin-dUTP Nick End Labeling

Cell apoptosis was also detected using terminal deoxynucleotidyl transferase biotin-dUTP nick end labeling (TUNEL) Apoptosis Assay Kits (C1086, Beyotime) in line with the manufacturer's specifications. In a nutshell, the cells were first fixed and permeated. Next, cells were stained with 50 *μ*l TUNEL solution for 45–60 min at room temperature, washed with 250–500 *μ*l PBS, and captured under a fluorescence microscope (OLYMPUS, CKX41, Japan).

### 2.12. Western Blot

The total cellular protein was extracted using radioimmunoprecipitation assay (RIPA) lysis buffer. The extracted protein concentration was quantified using a BCA Protein Assay Kit (Servicebio, Wuhan, China). The protein was separated using SDS-PAGE. Target proteins were transferred onto the polyvinylidene fluoride membranes. 5% skim milk powder was used to block the transferred membranes, which were then incubated with anti-CSDE1 (Abcam, ab201688) and anti-GAPDH (Abcam, ab181603) primary antibodies at 4°C overnight. After rinsing with Tris-Buffered Saline with Tween® 20, the membranes were incubated with secondary antibodies (BOSTER, BA1056) at 25°C for 1 hour. Finally, target protein signals were visualized after enhanced chemiluminescence.

### 2.13. Dual-Luciferase Reporter Assay

The relationships among LINC01234, miR-525-5p, and CSDE1 were analyzed using dual-luciferase reporter assay. LINC01234 wild-type (WT), LINC01234 mutated-type (MUT), CSDE1-WT, and CSDE1-MUT vectors were all constructed by Synbio Technologies. After cotransfection for 48 h, luciferase activity was detected using the Dual-Luciferase Reporter Assay System (Promega).

### 2.14. Fluorescence In Situ Hybridization

The cellular localization of LINC01234 in BC cells was determined using fluorescence in situ hybridization (FISH) assay. Both LINC01234 (5′-CACATGCTAAGTGGTGGGTGGGGTGAG-3′) and U6 (5′-AACGCTTCACGAATTTGCGT-3′) probes were acquired from RiboBio (Guangzhou, China). Cy3-labeled probes were hybridized with BC cells according to the explanatory memorandum [30]. The nuclei were stained with DAPI, and images were captured using a fluorescence microscope (OLYMPUS, CKX41, Japan).

### 2.15. RNA Immunoprecipitation Assay

RIP was performed with an EZ-Magna RIP Kit (Millipore, USA) according to the product manual. The AGO2 antibody was used for RIP. In brief, 1 × 10^6^ cells were lysed in 1 ml lysis buffer containing 1% protease inhibitor and RNase inhibitor on ice for 10 min. These cell lysates were centrifuged at 12000 *g* for 20 min at 4°C, and the supernatant was collected. Then, 100 *μ*l of supernatant was retained as input control. Following this, 500 *μ*l of protein A+G beads were incubated with 5 *μ*g of IgG or AGO2 antibody (Abcam, ab156870) and 450 *μ*l supernatant at 4°C for 12 h on a shaking table at 15 rpm/min. The coimmunoprecipitated RNAs were detected by qRT-PCR.

### 2.16. RNA Pull-Down Assay

The RNA pull-down assay was performed as previously reported [31]. Briefly, 200 nM biotinylated miR-525-5p, 100 nM miR-525-5p without biotin labeling, 100 nM biotinylated antisense of miR-525-5p, and 100 nM antisense of miR-525-5p without biotin labeling (GenePharma) were transfected into MCF-7 cells by Lipofectamine™ 3000 reagent (Invitrogen). After 24 h, 1 × 10^7^ cells were collected and lysed in the lysis buffer. Further, 500 *μ*l of cell lysate was incubated with 500 *μ*l of washed streptavidin magnetic beads (Life Technologies) for 2 h at 37°C. The beads were washed, and RNA was extracted with the TRIzol reagent. The coprecipitated RNA was analyzed using qRT-PCR.

### 2.17. Statistical Analysis

All data were calculated with GraphPad Prism software 8 (GraphPad Software, San Diego, CA, USA) and SPSS 22.0 software (IBM Corp., Armonk, NY, USA). The data are exhibited as mean ± SD. Student's *t*-test and one-way ANOVA were used for comparisons between two groups and multiple groups, respectively. Pearson's chi-square test was used to analyze correlations. Each experiment was performed at least in triplicate, and *p* < 0.05 was considered statistically significant.

## 3. Results

### 3.1. LINC01234 Was Upregulated and Correlated with a Poor Prognosis of BC

**Figure 1:**
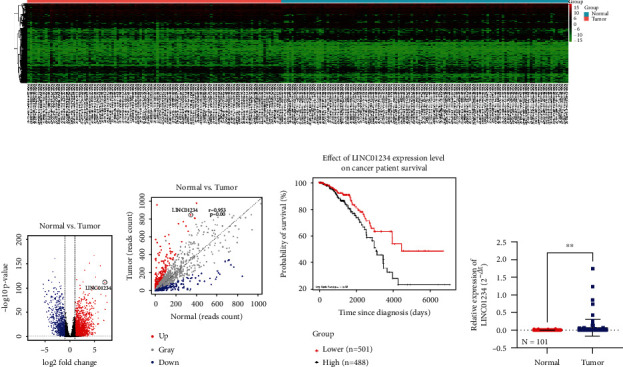
LINC01234 is increased and correlated with poor prognosis in breast cancer (BC). (a, b) A heat map and volcano plot chart showing the distribution of the DEG TCGA datasets. Green: downregulated genes; red: upregulated genes. (c) The correlation of gene expression between tumor and normal groups. (d) The patients with high LINC01234 expression have poor overall survival (OS). (e) The expression level of LINC01234 using quantitative real-time reverse transcription polymerase chain reaction in BC tissues (*n* = 101). ^∗∗^*p* < 0.01.

The expressed lncRNAs based on TCGA database were first analyzed, and 3034 DElncRNAs were identified using the DESeq2 package assuming cutoff values of logFC > 2 and *p* < 0.05. These included 2151 upregulated and 883 downregulated DElncRNAs (Figures 1(a) and 1(b)). The correlation between gene expression in the tumor and normal groups was analyzed using R software (Figure 1(c)). Based on bioinformatic analyses, LINC01234 was picked as the candidate gene for further investigation. Survival analysis to analyze the role of LINC01234 in BC revealed that patients with high LINC01234 expression had poorer overall survival (OS) (Figure 1(d)). To explore the clinicopathological implications of LINC01234, we collected 101 paired BC tissue samples and evaluated the expression levels of LINC01234 using qRT-PCR. LINC01234 was found to be increased in BC tissues (Figure 1(e)). Based on the median value of LINC01234, patients were divided into low- and high-expression groups. We found that LINC01234 expression level was positively correlated with the tumor size of BC (Table 2). Thus, our results indicated that LINC01234 may contribute to tumorigenesis in BC, an association that has hitherto remained unexplored.

### 3.2. LINC01234 Functioned as an Oncogene in BC Cells

**Figure 2:**
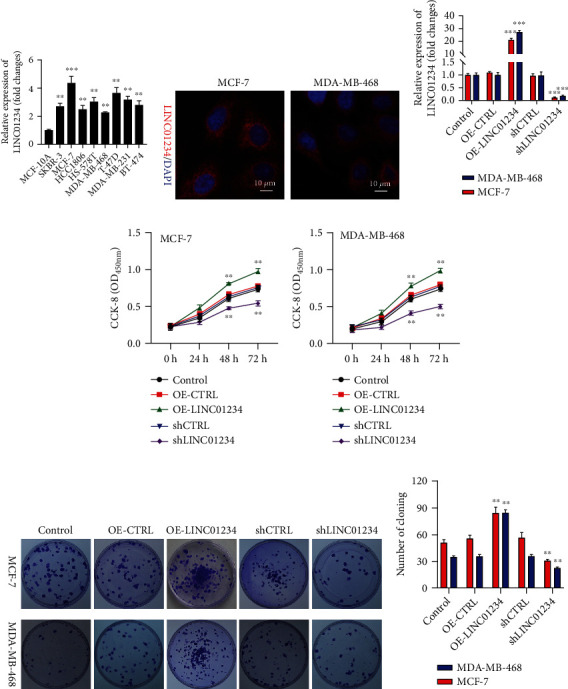
Inhibition of LINC01234-suppresses BC cell proliferation. (a) LINC01234 expression in breast cancer (BC) cells is notably higher than that in MCF-10A. (b) Fluorescence in situ hybridization shows that LINC01234 is mainly expressed in the cytoplasm. (c) Quantitative real-time reverse transcription polymerase chain reaction demonstrates that LINC01234 is successfully inhibited or overexpressed in BC cells. (d, e) CCK-8 assay verifies that the knockdown of LINC01234 notably suppresses cell growth, while overexpression of LINC01234 promotes it. (f, g) Colony formation assay verifies that knockdown of LINC01234 notably suppresses colony-forming activity and overexpression of LINC01234 promotes it. ^∗∗^*p* < 0.01; ^∗∗∗^*p* < 0.001.

**Figure 3:**
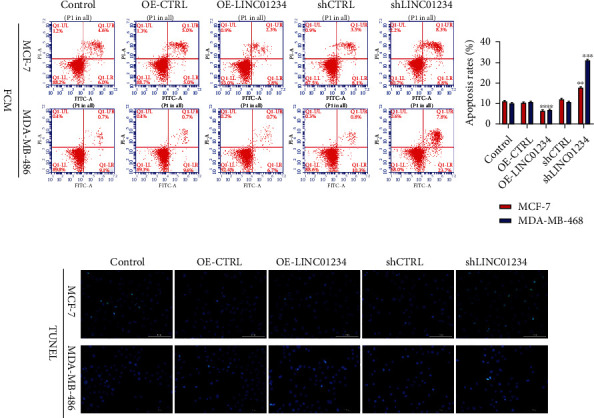
Inhibition of LINC01234 promotes breast cancer cell apoptosis. (a) Flow cytometry results display that downregulation of LINC01234 markedly promotes cell apoptosis. Meanwhile, overexpression of LINC01234 shows the opposite effect. (b) Terminal deoxynucleotidyl transferase biotin-dUTP nick end labeling staining results show that downregulation of LINC01234 markedly promotes cell apoptosis. Meanwhile, overexpression of LINC01234 shows the opposite effects. ^∗∗^*p* < 0.01; ^∗∗∗^*p* < 0.001.

To determine the role of LINC01234 *in vivo*, we detected LINC01234 expression levels in eight BC cell lines and MCF-10A. The LINC01234 expression in BC cells was notably higher than that in normal cell MCF-10A (Figure 2(a)). To select the most representative cell lines for the study of LINC01234's effects, we chose MDA-MB-468 (with the lowest LINC01234 expression) and MCF-7 cells (with the highest) in the subsequent experiments. Furthermore, FISH results confirmed that LINC01234 was mainly expressed in the cytoplasm (Figure 2(b)). We used RNA interference or overexpression plasmids to change LINC01234 expression in BC cells. The results of qRT-PCR revealed that LINC01234 was successfully inhibited or overexpressed in BC cells (Figure 2(c)). Cell growth assays verified that the knockdown of LINC01234 notably suppressed cell growth and colony-forming activity, while the overexpression of LINC01234 promoted them (Figures 2(d)–2(g)). Flow cytometry and terminal deoxynucleotidyl transferase biotin-dUTP nick end labeling (TUNEL) staining demonstrated that downregulation of LINC01234 markedly promoted cell apoptosis. Meanwhile, overexpression of LINC01234 showed the opposite effects (Figures 3(a) and 3(b)). All these outcomes suggest that LINC01234 promotes cell proliferation and inhibits cell apoptosis, functioning as an oncogene in BC cells.

### 3.3. LINC01234 Directly Interacted with miR-525-5p as a Sponge in BC Cells

**Figure 4:**
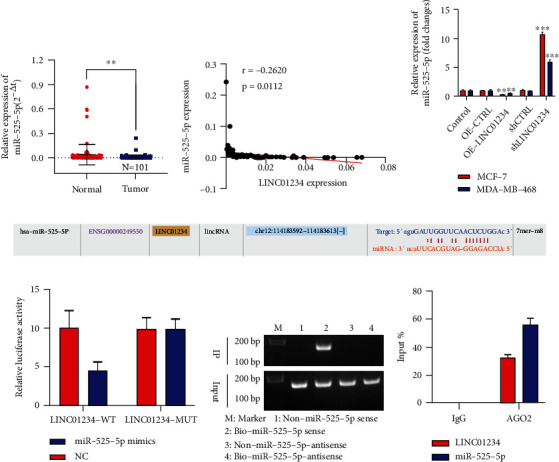
LINC01234 directly interacts with miR-525-5p as a sponge in breast cancer cells. (a) qRT-PCR results show that miR-525-5p is downregulated in breast cancer (BC) tissues. (b) miR-525-5p expression levels are negatively correlated with those of LINC01234. (c) Overexpression of LINC01234 significantly attenuates miR-525-5p expression in BC cells, while downregulation LINC01234 promotes miR-525-5p expression. (d) The potential binding sites of LINC01234 within the miR-525-5p sequence. (e) The luciferase activity is significantly suppressed on cotransfecting LINC01234-WT and miR-525-5p mimics in cells. (f) RNA pull-down results show that miR-525-5p can band with LINC01234. (g) RNA immunoprecipitation results showed that LINC01234 and miR-525-5p are specifically enriched in the AGO2 antibody immunoprecipitated complexes. ^∗∗^*p* < 0.01; ^∗∗∗^*p* < 0.001.

As we already know, lncRNA distributed in the cytoplasm can function as sponges, adsorbing miRNA and liberating the corresponding miRNA-targeted mRNA transcripts. Accordingly, we predicted the target miRNAs of LINC01234 using lncBaseV2.0, miRcode, and StarBase. miR-525-5p was jointly identified as one of the potential target miRNAs of LINC01234. First, we found that the levels of miR-525-5p were decreased in BC tissues (Figure 4(a)) and were negatively correlated with the expression levels of LINC01234 (Figure 4(b)). Moreover, overexpressed LINC01234 significantly attenuated miR-525-5p expression, while downregulated LINC01234 promoted miR-525-5p expression (Figure 4(c)). According to public databases, the potential binding sites of LINC01234 are found within the miR-525-5p sequence (Figure 4(d)). Therefore, we structured the LINC01234 luciferase reporter containing the putative wild type or mutant type. Luciferase activity was significantly suppressed on cotransfecting with LINC01234-WT and miR-525-5p mimics in cells (Figure 4(e)). Furthermore, RNA pull-down results showed that miR-525-5p could band with LINC01234 (Figure 4(f)). In addition, we performed RIP experiments to pull down the complex using AGO2 antibodies and carried out qRT-PCR for miR-525-5p and LINC01234. The results revealed that miR-525-5p and LINC01234 were specifically enriched in the AGO2 antibody-immunoprecipitated complexes (Figure 4(g)). Consequently, our findings indicated that LINC01234 could directly bind to miR-525-5p in a structural and functional manner.

### 3.4. miR-525-5p Functioned as an Antioncogene in BC Cells

**Figure 5:**
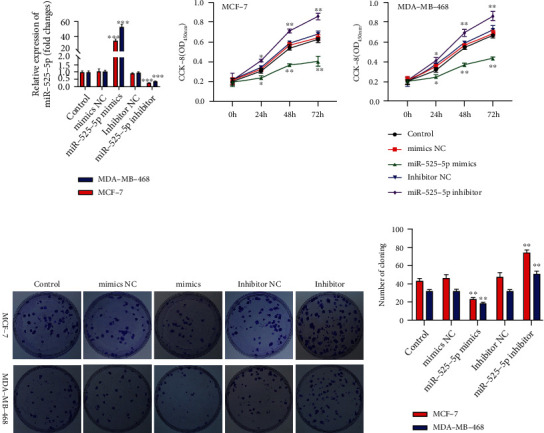
Upregulation of miR-525-5p suppresses breast cancer cell proliferation. (a) Quantitative real-time reverse transcription polymerase chain reaction shows that miR-525-5p is successfully inhibited or overexpressed in MCF-7 and MDA-MB-468 cells. (b–d) CCK-8 and colony formation assays verify that overexpression of miR-525-5p notably suppresses cell growth and colony-forming activity, while suppression of LINC01234 promotes it. ^∗∗^*p* < 0.01; ^∗∗∗^*p* < 0.001.

**Figure 6:**
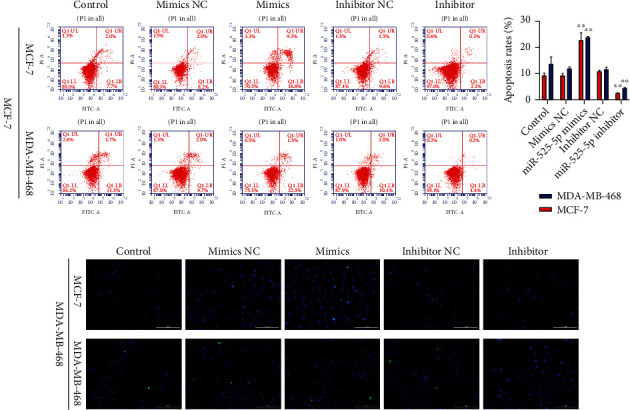
Overexpression of miR-525-5p promotes breast cancer cell apoptosis. (a, b) Flow cytometry and terminal deoxynucleotidyl transferase biotin-dUTP nick end labeling staining results display that upregulation of miR-525-5p markedly promotes cell apoptosis. Conversely, downregulation of miR-525-5p shows the opposite effect. ^∗∗^*p* < 0.01.

On qRT-PCR, miR-525-5p was successfully inhibited or upregulated in BC cells after transfection with miR-525-5p mimics and inhibitors, respectively (Figure 5(a)). Cell growth assays verified that the upregulation of miR-525-5p notably suppressed the growth and colony-forming activities of the cell, while suppression of miR-525-5p promoted cell growth and colony-forming activities (Figures 5(b)–5(d)). Flow cytometry and TUNEL staining revealed that the upregulation of miR-525-5p dramatically promoted cell apoptosis. Conversely, downregulation of miR-525-5p showed the opposite effects (Figures 6(a) and 6(b)). These outcomes implied that miR-525-5p functioned as a tumor suppressor in BC and was sponged by LINC01234.

### 3.5. CSDE1 Was a Direct Target of miR-525-5p

**Figure 7:**
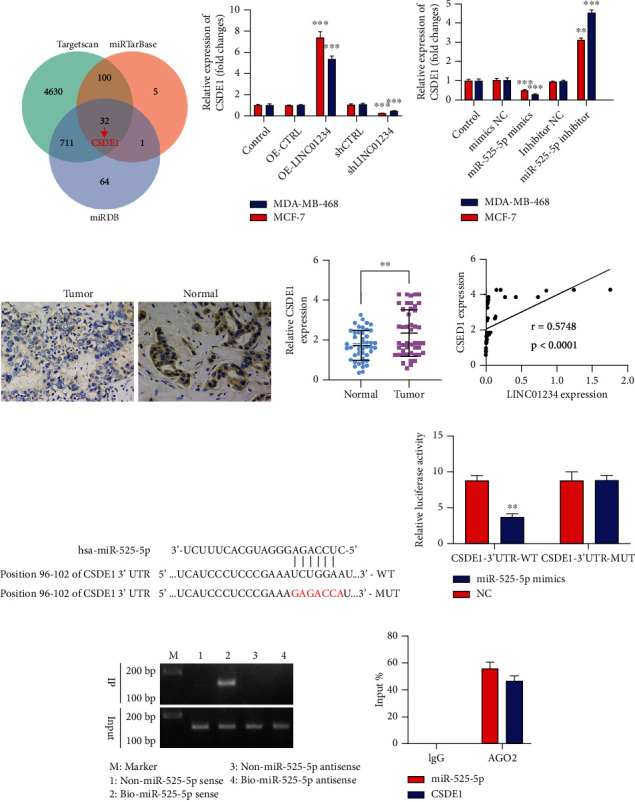
CSDE1 is a target gene of miR-525-5p. (a) On miRDB, miRTarBase, and TargetScan, 32 overlapping target mRNA are identified, and CSDE1 is chosen as the gene potentially downstream of miR-525-5p. (b) Quantitative real-time reverse transcription polymerase chain reaction (qRT-PCR) analyses validate that CSDE1 is increased and reduced after transfection with LINC01234 overexpression plasmid and small interfering RNA in breast cancer (BC) cells. (c) qRT-PCR analysis of the expression of CSDE1. (d) Immunohistochemical (IHC) analysis of the expression of CSDE1 in BC tissues and normal tissues. (e) Quantification of IHC results. (f) Correlation between CSDE1 expression and LINC01234 expression in BC tissues. (g) The potential binding sequences between miR-525-5p and 3′UTR of CSDE1. (h) miR-525-5p mimics cotransfected with CSDE1-WT notably reduce luciferase activity. (i) RNA pull-down results show that miR-525-5p can band with the 3′UTR of CSDE1. (j) RNA immunoprecipitation results reveal that CSDE1 and miR-525-5p are specifically enriched in the AGO2 antibody immunoprecipitated complexes. ^∗∗^*p* < 0.01; ^∗∗∗^*p* < 0.001.

To confirm whether LICN01234 functions as a ceRNA of miR-525-5p and liberates the expression levels of downstream target genes, we predicted the potential target mRNA of miR-525-5p by employing miRDB, miRTarBase, and TargetScan. A total of 32 overlapping target mRNA were identified, and *CSDE1* was chosen as the gene potentially downstream of miR-525-5p (Figure 7(a)). qRT-PCR analyses validated that CSDE1 was increased and reduced after transfection with LINC01234 overexpression plasmid and small interference RNA (siRNA) in BC cells (Figure 7(b)), and CSDE1 expression was decreased by miR-525-5p mimics and upregulated by a miR-525-5p inhibitor (Figure 7(c)). Furthermore, we analyzed CSED1 protein expression in BC and the tumor-adjacent normal tissues using IHC and found that CSDE1 levels were significantly increased in BC than in normal tissues (Figures 7(d) and 7(e)), and its levels were positively correlated with LINC01234 expression (Figure 7(f)). The predicted binding sequences between miR-525-5p and 3′UTR region of CSDE1 are shown in Figure 7(g). The luciferase reporter assay revealed that miR-525-5p mimics cotransfected with CSDE1-WT notably reduced the relative luciferase activity in cells (Figure 7(h)). Furthermore, RNA pull-down results showed that miR-525-5p could band with CSDE1 (Figure 7(i)). In addition, RIP and qRT-PCR for CSDE1 and miR-525-5p revealed that miR-525-5p and CSDE1 were specifically enriched in the AGO2 antibody immunoprecipitated complexes (Figure 7(j)). These findings indicated that miR-525-5p could directly bind with CSDE1.

### 3.6. *CSDE1* Functioned as an Oncogene in BC Cells

**Figure 8:**
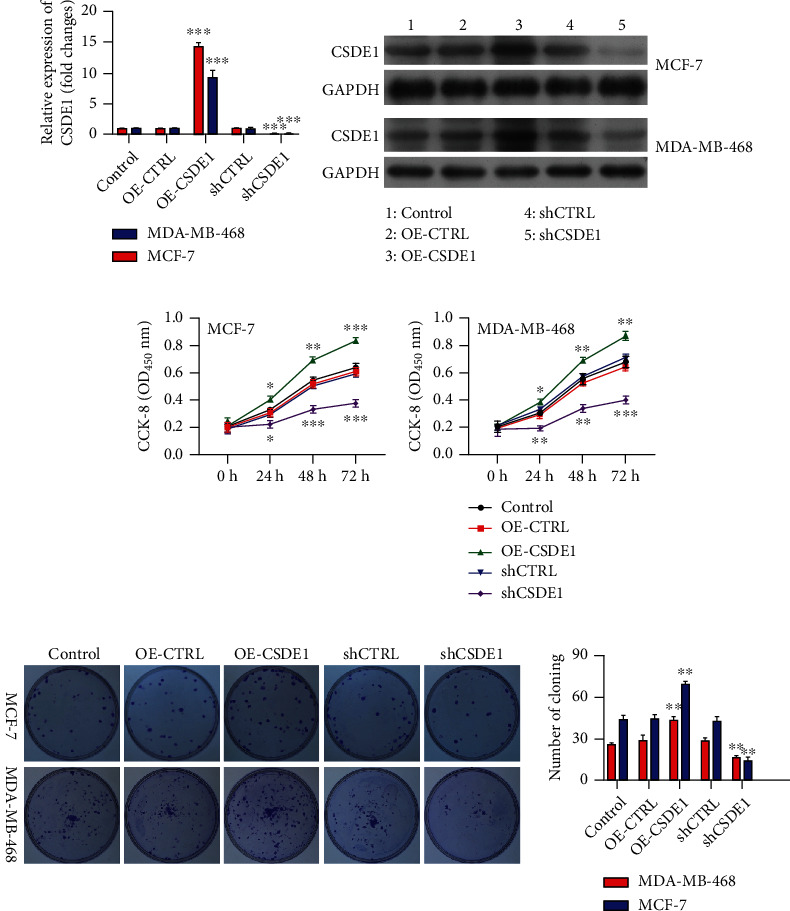
Inhibition of CSDE1 suppresses breast cancer cell proliferation. (a, b) Quantitative real-time reverse transcription polymerase chain reaction and Western blot results show that CSDE1 is successfully inhibited or overexpressed in MCF-7 and MDA-MB-468 cells. (c, d) CCK-8 and colony formation assays verify that knockdown of CSDE1 notably suppresses cell growth and colony-forming activity, while overexpression of CSDE1 promotes them. ^∗∗^*p* < 0.01; ^∗∗∗^*p* < 0.001.

**Figure 9:**
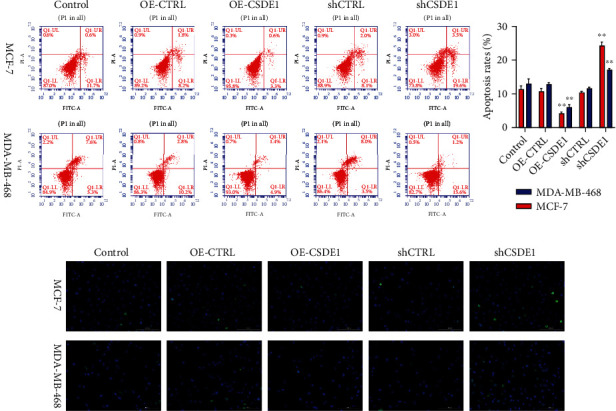
Suppression of CSDE1 induces breast cancer cell apoptosis. (a, b) Flow cytometry and terminal deoxynucleotidyl transferase biotin-dUTP nick end labeling staining show that downregulation of CSDE1 markedly promotes cell apoptosis, while overexpression of CSDE1 has the opposite effect. ^∗∗^*p* < 0.01.

We used siRNA or overexpression plasmids to inhibit or overexpress *CSDE1* in BC cells, respectively (Figures 8(a), 8(b), and S1A). Cell growth assays verified that the knockdown of *CSDE1* notably suppressed cell growth and colony-forming activity, while overexpression of *CSDE1* promoted them (Figures 8(c) and 8(d)). Flow cytometry and TUNEL staining demonstrated that downregulation of *CSDE1* markedly promoted cell apoptosis. The overexpression of *CSDE1* showed the opposite effects (Figures 9(a) and 9(b)). All these outcomes suggested that *CSDE1* promoted cell proliferation and inhibited cell apoptosis, thereby functioning as an oncogene in BC cells.

### 3.7. LICN01234 Promoted BC Progression by Relieving the Repression of miR-525-5p on CSDE1

**Figure 10:**
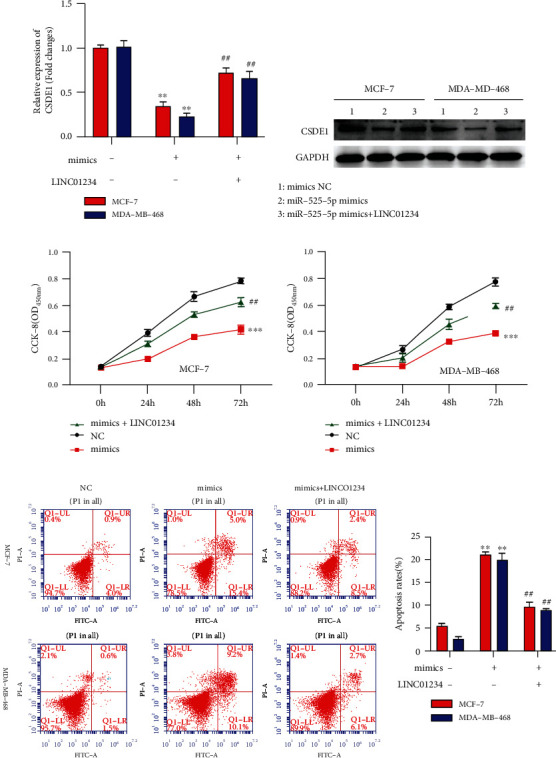
LICN01234 promotes breast cancer progression by relieving the repression of miR-525-5p on CSDE1. (a, b) In the rescue experiments, CSDE1 is significantly decreased by miR-525-5p mimics, which are rescued by LINC01234 overexpression. (c, d) Overexpression of miR-525-5p inhibits cell proliferation, while overexpression of LINC01234 can reverse miR-525-5p mimic-mediated suppression of cell proliferation. (e) Overexpression of miR-525-5p promotes cell apoptosis, while overexpression of LINC01234 can reverse miR-525-5p mimic-mediated promotion of cell apoptosis. ^∗∗^*p* < 0.01 vs. NC group; ^##^*p* < 0.01 vs. miR-525-5p mimics group.

The biological functions of LINC01234 were verified in the above experiments. However, it was unclear how LINC01234 worked with miR-525-5p and CSDE1 together. In the rescue experiments, CSDE1 was significantly decreased by miR-525-5p mimics and rescued by LINC01234 overexpression (Figures 10(a), 10(b), and S1B). An increase in miR-525-5p inhibited cell growth and stimulated cell apoptosis, while overexpression of LINC01234 reversed miR-525-5p mimics-mediated cellular phenotypes (Figures 10(c)–10(e)). These results implied that LICN01234 competitively combined with miR-525-5p to relieve the repression of CSDE1, thus propelling BC progression.

## 4. Discussion

Breast cancer is a complex and heterogeneous cancer. In recent years, several lncRNAs have piqued the interest of researchers and have been demonstrated to play vital functions in BC. However, lncRNAs are massive in number, making those that have been reported in BC merely the “the tip of the iceberg.” In this study, we used TCGA database and filtrated out a number of aberrantly expressed lncRNAs. LINC01234 goaded our interest since it was upregulated in BC and related to poor prognosis in BC patients.

LINC01234, located on chromosome 12, plays an active role in several cancers. Suppression of LINC01234 expression was found to restrain liver cancer progression via the mediation of the miR-513a-5p/USP4/TGF-*β* axis [32]. LINC01234 was also shown to regulate the progression of clear cell renal cell carcinoma cells by HIF-2a pathways [33]. In our study, LINC01234 was highly expressed in 101 pairs of BC patients' tissues and BC cells and was significantly associated with BC tumor size. LINC01234 promoted BC cell growth and inhibited cell apoptosis. These outcomes imply that LINC01234 can act as an oncogene to accelerate BC progression and may be a potential therapeutic target for BC. Besides, LINC01234 markedly accelerated cell migration and proliferation of MDA-MB-231 cells *in vitro* and inhibited neoplasia *in vivo* [19]. LINC01234 was also shown to promote cell proliferation and tumor metastasis in triple-negative breast cancer (TNBC) [34]. Here, we paid close attention to the role of LINC01234 in cell proliferation and found that LINC01234 overexpression promoted cell growth *in vitro*. The role of LINC01234 in cell migration and invasion of BC cells needs to be further investigated.

Depending on their localization, lncRNAs in the cytoplasm bear miRNA-complementary sites as a sponge and regulate gene expression [35]. In this study, FISH results showed that LINC01234 was mainly expressed in the cytoplasm, suggesting that it functions as a miRNA sponge. Recent studies showed that LINC01234 promotes cancer development as a miRNA sponge and can regulate multiple miRNAs, including miR-27b-5p [17], miR-525-5p [34], miR-106b [36], miR-193a-5p [37], miR-433 [38], miR-340-5p and miR-27b-3p [39], miR-1284 [40], miR-637 [41], miR-140 [42], miR-642a-5p [43], and miR-204-5p [44]. Furthermore, miR-525-5p has been found to act as a potential tumor suppressor miRNA in various cancers, such as colorectal cancer [45], chordoma [46], lung adenocarcinoma [47], non-small-cell lung cancer [22], triple-negative breast cancer [34], and thymoma and thymic carcinoma [23], among others. However, the role of miR-525-5p in BC remains scarcely known. Here, we predicted that LICN01234 would have binding sites for miR-525-5p, which has been reported as an antioncogene in several cancers. Our findings revealed that LICN01234 and miR-525-5p levels were inversely correlated in BC samples and confirmed the direct interaction between LICN01234 and miR-525-5p. We also observed that miR-525-5p could inhibit cell proliferation and facilitate cell apoptosis, thereby directly opposing the effects of LINC01234, further supporting the role of LINC01234 as an endogenous sponge for miR-525-5p.

Recent studies describe a complex interplay among lncRNAs, miRNAs, and mRNAs. Abundant lncRNAs, as ceRNAs or “sponges” of miRNAs, alter the stability and translation of miRNAs-target-mRNAs and interfere with signaling pathways [48, 49]. We predicted and verified that miR-525-5p targeted binding to CSDE1. CSDE1 was significantly overexpressed in BC cells, where they enhanced cell growth and inhibited apoptosis. In the rescue experiments, CSDE1 levels were significantly reduced when miR-525-5p was overexpressed but were soon rescued by the addition of pcDNA-LINC01234, suggesting that LINC01234 counteracted the inhibition of miR-525-5p on CSDE1, a phenomenon that was further confirmed in cellular functional experiments. All these results indicate that LINC01234 can be used as a ceRNA to competitively adsorb miR-525-5p, thus weakening the inhibition of miR-525-5p on the downstream target gene CSDE1, thereby enhancing the oncogenic effect of CSDE1, which may have profound effects in BC development.

CSDE1, a conserved RNA binding protein, is also called upstream of N-Ras (UNR); it has a high capacity for binding with RNA [50, 51]. It can not only decide the translation initiation and expression of certain mRNAs but also determine the stability and abundance of these mRNAs [52]. Besides, CSDE1 has been recorded to facilitate tumor progression by driving a posttranscriptional program [50, 53]. miR-212 and miR-132 cluster can hinder thyroid cancer progression by directly targeting CSDE1 [54]. The above evidence is in line with our results. We found that LINC01234 had tumor-promoting effects in BC by attenuating the suppression of miR-525-5p on CSDE1. Duan et al. analyzed the target genes of CSDE1 in BC. A total of 826 CSDE1-associated target genes were mainly associated with ribosomes, transcriptional misregulation, and metabolic pathways [55]. Another investigation discovered that CSDE1 could regulate the expression of c-Myc, Rac-1, PTEN, and vimentin in colorectal cancer [56]. Besides, CSDE1 was also verified to have tumor-promoting effects in melanoma by regulating the translation of vimentin and Rac-1 [53]. Moreover, Tian et al. [57] revealed that UNR facilitated the migration of glioma cells by binding to the 3′UTR of RPL9 and PTEN. These findings imply that CSDE1 participates in cancer cell signaling pathways. We speculate that LINC01234 may ultimately function via the miR-525-5p/CSDE1 pathway in BC, given CSDE1-mediated downstream complex regulatory networks. Therefore, further studies into these pathways are warranted to thoroughly investigate the pathogenesis of BC.

## 5. Conclusion

Our research indicated that LINC01234 was increased and correlated with poor clinical outcomes in BC. LINC01234 enhanced cell growth and restrained cell apoptosis in BC. Moreover, LINC01234 functioned as a ceRNA to increase CSDE1 expression levels, by sponging miR-525-5p, causing BC progression. LINC01234 might be used as one of the potential biomarkers and therapeutic targets in BC.

## Figures and Tables

**Table 1:** The sequences of the primers used in quantitative real-time reverse transcription polymerase chain reaction.

Gene	Sequence (5′-3′)	Product length (bp)
GAPDH.F	TGTTCGTCATGGGTGTGAAC	154
GAPDH.R	ATGGCATGGACTGTGGTCAT	
LINC01234.F	TCAGCAACTGCTAACAGCGA	195
LINC01234.R	GTGTGACTTCGGAGGGAGTG	
CSDE1.F	AAACCAGAATGACCCATTGCC	152
CSDE1.R	ATTTGTCACGTCGGTCTGTTG	
U6.F	CTCGCTTCGGCAGCACA	96
U6.R	AACGCTTCACGAATTTGCGT	
hsa-miR-525-5p.R	CTCAACTGGTGTCGTGGA	
hsa-miR-525-5p.RT	CTCAACTGGTGTCGTGGAGTCGGCAATTCAGTTGAGAGAAAGTG	
hsa-miR-525-5p.F	ACACTCCAGCTGGGCTCCAGAGGGATGCACT	

**Table 2:** The correlation between LINC01234 expression and the clinical and pathological features of patients with breast cancer.

Clinical characteristics	Number of cases (*N* = 101)	LINC01234 expression		*p* value
		High (*N* = )	Low (*N* = )	
*Age (years)*				0.607
≤50	26	12	14	
>50	75	39	36	
*Gender*				0.31
Male	1	0	1	
Female	100	51	49	
*Tumor size (cm)*				0.013^∗^
≤2	50	19	31	
>2	51	32	19	
*Pathologic stage*				0.636
Stage I+II	83	41	42	
Stage III+IV	18	10	8	
*Lymph node metastasis*				0.778
Yes	37	18	19	
No	64	33	31	
*ER*				0.119
Negative	25	16	9	
Positive	76	35	41	
*PR*				0.352
Negative	41	23	18	
Positive	60	28	32	
*HER2*				0.613
Negative	28	13	15	
Positive	73	38	35	

## Data Availability

The data and study materials that support the findings of this study will be available to other researchers from the corresponding authors on reasonable request.

## Additional Points

*Highlights*. (i) LINC01234 is upregulated and associated with poor prognosis in BC. (ii) LINC01234 promotes proliferation and inhibits apoptosis of BC cells. (iii) LINC01234 serves as a sponge for miR-525-5p. (iv) CSDE1 is a target of miR-525-5p.

## Supplementary Materials

Supplementary materials Figure S1: quantification of protein expression. (A) The protein expression in MCF-7 and MDA-MB468 cells after transfection with CSDE1 overexpression plasmids or small interfering RNA, ^∗∗^*p* < 0.01. (B) The protein expression in MCF-7 and MDA-MB-468 cells with different treatment, ^∗∗^*p* < 0.01 vs. NC and ^##^*p* < 0.01 vs. mimics.
