# Supplementary material for: LINC01234 Accelerates the Progression of Breast Cancer via the miR-525-5p/Cold Shock Domain-Containing E1 Axis
Source: Dis Markers. 2022 Jul 25;2022:6899777. doi: 10.1155/2022/6899777 (PMC9343190; doi:10.1155/2022/6899777)
Supplement: Supplementary materials — Figure S1: quantification of protein expression. (A) The protein expression in MCF-7 and MDA-MB468 cells after transfection with CSDE1 overexpression plasmids or small interfering RNA, ∗∗p < 0.01. (B) The protein expression in MCF-7 and MDA-MB-468 cells with different treatment, ∗∗p < 0.01 vs. NC and ##p < 0.01 vs. mimics. [file 6899777.f1.pdf]

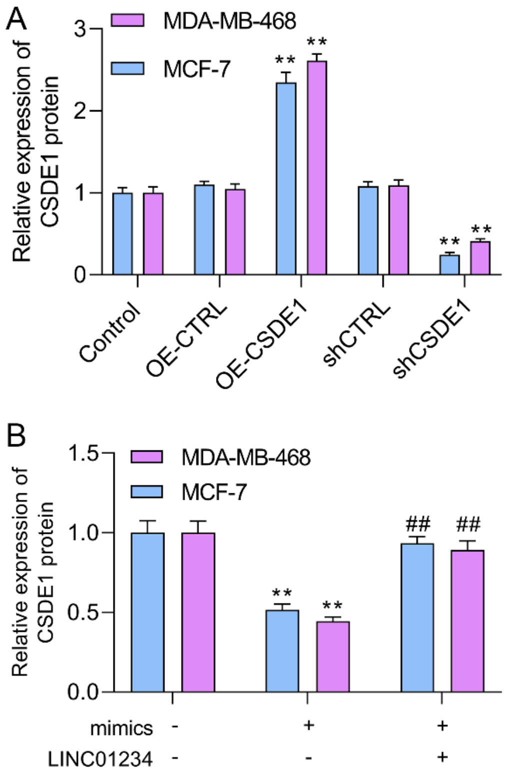

Figure S1. Quantification of protein expression. (A)The protein expression in MCF-7 and MDA-MB-468 cells after transfection with CSDE1 overexpression plasmids or small interfering RNA, ** $p < 0.01$. (B) The protein expression in MCF-7 and MDA-MB-468 cells with different treatment, ** $p < 0.01$ vs NC, ## $p < 0.01$ vs mimics.
